# On the Effect of Lattice Topology on Mechanical Properties of SLS Additively Manufactured Sheet-, Ligament-, and Strut-Based Polymeric Metamaterials

**DOI:** 10.3390/polym14214583

**Published:** 2022-10-28

**Authors:** Aliaa M. Abou-Ali, Dong-Wook Lee, Rashid K. Abu Al-Rub

**Affiliations:** 1 Advanced Digital & Additive Manufacturing Center, Khalifa University, Abu Dhabi P.O. Box 127788, United Arab Emirates; 2 Advanced Material Research Centre, Technology Innovation Institute, Abu Dhabi P.O. Box 9639, United Arab Emirates; 3 Mechanical Engineering Department, Khalifa University, Abu Dhabi P.O. Box 127788, United Arab Emirates

**Keywords:** additive manufacturing, triply periodic minimal surfaces, lattices, selective laser sintering, architected materials

## Abstract

Cellular lattices with architectural intricacy or metamaterials have gained a substantial amount of attention in the past decade due to the recent advances in additive manufacturing methods. The lattice topology controls its physical and mechanical properties; therefore, the main challenge is selecting the appropriate lattice topology for a desired function and application. In this work, we comprehensively study the topology–property relationship of three classes of polymer metamaterials based on triply periodic minimal surfaces (TPMS) of sheet/shell and ligament types, and other types of well-known strut-based lattices. The study uses a holistic approach of designing, additive manufacturing, microstructural characterization, and compressive uniaxial mechanical testing of these polymer lattices that are 3D-printed using the laser powder bed fusion technique known as selective laser sintering (SLS). In total, 55 lattices with different topologies and relative densities were 3D-printed and tested. Printing quality was assessed using scanning electron microscopy and micro-computed tomography. The extracted mechanical properties of elastic modulus, yield strength, plateau strength, and energy absorption are thoroughly compared between the different lattice architectures. The results show that all the investigated ligament-based TPMS polymer lattices exhibit bending-dominated elastic and plastic behavior, indicating that they are suitable candidates for energy absorbing applications. The sheet-based TPMS polymer lattices, similarly to the well-known Octet-Truss lattice, exhibited an elastic stretching-dominated mode of deformation and proved to have exceptional stiffness as compared to all other topologies, especially at low relative densities. However, the sheet-based TPMS polymer lattices exhibited a bending-dominated plastic behavior which is mainly driven by manufacturing defects. Overall, however, sheet-based TPMS polymer lattices exhibited the best mechanical properties, followed by strut-based lattices and finally by ligament-based TPMS lattices. Finally, it is depicted that at high relative densities, the mechanical properties of lattices of various architectures tend to converge, which implies that the topological effect is more significant at low relative densities. Generally, this study provides important insights about the selection of polymer mechanical metamaterials for various applications, and shows the superiority of TPMS-based polymer metamaterials as compared to several other classes of polymer mechanical metamaterials.

## 1. Introduction

Architected interconnected and cellular networks of solid struts, plates, or shells that form edges and faces of cells are known as metamaterials or latticed materials [1,2,3,4]. Intricate cellular solid structures are originally found in nature such as bones, bird wings, and wood and have inspired a plethora of studies due to the multi-functional properties that they can provide with reduced weight. Cellular solids with a length scale much smaller than the size of the structure can act as a material with its own set of effective properties and have desirable mechanical properties when compared to their fully dense solid parts, including enhanced mechanical energy absorption, high stiffness-to-weight ratio, and heat and mass transfer control [5]. Thus, cellular solids are used in many applications, such as energy absorption, heat exchangers and sinks, thermal insulation, acoustic and vibration damping, lightweight structures, tissue scaffolds, catalytic substrates, filtering, etc. [6,7,8,9,10,11,12,13,14]. However, there is a coupling between their mechanical properties and their topology, relative density (i.e., which is the ratio of the density of the cellular material to that of its solid counterpart), and properties of the base material; thus, the challenge is to achieve enhanced properties with low density [15].

The most common types of cellular solids are stochastic foams that are considered a subset of cellular solids and were studied long before the attention shifted to architected or periodic cellular solids or lattices (mostly known these days as metamaterials). However, the mechanical behavior of foams could be less reliable and hard to predict due to their random structures and possible extensive flaws and defects from manufacturability. To overcome such problems, lattice materials are designed to have an accurate estimate of their mechanical properties and consist of periodic unit cells [1,16], where their mechanical behavior is mainly affected by the properties of the constituent material and geometrical features (e.g., topology, unit cell size, relative density). It is worth noting that by fixing the base material, morphology, unit cell size, and relative density, the mechanical properties primarily depend on the lattice topology of the unit cell. The main challenge in adapting lattices when it comes to engineering design is choosing the appropriate lattice topology from a plethora of lattice types that can provide the required mechanical properties to maximize the mechanical performance while reducing weight. Thus, understanding the material–topology–property relationship that reveals the mechanical behavior as a function of the relative density and the exhibited lattice topology is desirable to allow for the design of end-use components with enriched performance [17]. Most of the common structures of these cellular lattices are generated by creating computer-aided design (CAD) skeletons with sharp joints and edges. These lattice structures have straight beam-like struts that could create stress concentrations at the nodes, which might result in multi-axial stresses and progressive failures. In this regard, studies shifted towards lattice architectures that are mathematically derived, such as triply periodic minimal surfaces (TPMS)-based lattices [18,19,20,21,22,23,24,25,26,27,28,29,30,31,32,33,34,35,36,37,38,39,40,41,42,43,44,45,46]. TPMS have smooth surfaces, have no sharp corners or edges, and split the space into two or more infinite and non-intersecting domains. TPMS have been observed and inspired by nature, such as in soap films [36], block polymers [47], and butterfly wings [34]. From all of these interesting and unique features, Karcher and Polthier [30] stated that minimal surfaces have applications in many disciplines such as mathematics and natural science. TPMS can be optimally implemented in several engineering applications, such as fluid permeability [27], electrical and thermal conductivities [48], composites [49,50], lightweight structures [51,52], and tissue engineering [29,53]. TPMS can be used to create lattices either (1) in the form of sheet/shell-based structures by thickening the minimal surface or (2) in the form of ligament/solid network-based structures by solidifying one of the two volumes enclosed by the minimal surface [50].

Manufacturing complex lattice architectures, especially with small feature sizes, is a fundamental challenge. However, recent developments in advanced macro/micro additive manufacturing (AM) techniques can overcome the limitations of conventional manufacturing techniques. AM is versatile and has been used to produce functional intricate parts using a broad spectrum of materials such as composites, metals [9,21,22,39,40,45,51,52,54,55,56,57,58], polymers [19,28,32,35,37,46,59,60,61], and ceramics [13] with the aim of facilitating manufacturability, enhancing multi-functionality, reducing the overall weight, and saving materials. Demand for AM is progressively increasing due to their ability to fabricate complex architectures and custom-made parts. The common AM techniques for polymers are material jetting, photopolymerization, material extrusion, and powder bed fusion. The material used in these fabrication processes can be either liquid, powder, or solid [62].

The polymer laser powder bed fusion technique known as selective laser sintering (SLS) [63] is considered one of the most common polymeric AM techniques for polymer metamaterials [14,18,32,64]. However, to the best of our knowledge, there are no consistent comparison studies on the mechanical properties of SLS-fabricated lattices of different topologies and types, which is the focus of the current study. The literature includes many studies on TPMS lattices, such as scaffolds [20,39,43,44,65,66,67,68,69,70,71] and mechanical metamaterials being sheet-based [48] or TPMS as reinforcements [49,50]. Kadkhodapour et al. [28] investigated the mechanical behavior of polymeric Diamond ligament-based TPMS lattices at high relative densities manufactured using polyjet 3D printing. Montazerian et al. [35] studied polymeric Gyroid and Diamond ligament-based TPMS manufactured using fused filament fabrication (FFF) at high relative densities ranging between approximately 30% and 70%. Maskery et al. [32] investigated the mechanical behavior of one relative density only for polymeric Gyroid and Diamond ligament-based TPMS fabricated by SLS. Abueidda et al. [72] experimentally and computationally investigated the mechanical behavior of four topologies of sheet-based TPMS lattices (IWP, Primitive, Neovious, and Gyroid) fabricated by SLS. Al-Ketan et al. [5] studied metallic TPMS for both ligament- and sheet-based topologies using Maraging steel manufactured by laser powder bed fusion or selective laser melting (SLM). Abou-Ali et al. experimentally and computationally compared the mechanical behavior of sheet- and ligament-based TPMS lattices [18,73].

In the current literature, there are no systematic studies experimentally comparing polymeric cellular struts with ligament- and sheet-based TPMS lattices that are fabricated using SLS. In this regard, this paper compares the material–topology–property relationship of polymeric sheet-based TPMS, ligament-based TPMS, and cellular struts. This work extensively investigates the role of the topology in tuning the mechanical behavior by experimentally investigating and comparing the compressive mechanical properties of different sheet-based TPMS lattices, namely Schwarz Diamond, Schoen Gyroid, Schoen I-WP, and Fischer–Koch CY, with corresponding ligament-based lattices, and strut-based lattices with Gibson–Ashby (GA), Kelvin (K), Octet-Truss (OT), and Idealized Gyroid (IG) architectures at various relative densities.

## 2. Methods and Materials

### 2.1. Topological Design

**Figure 1:**
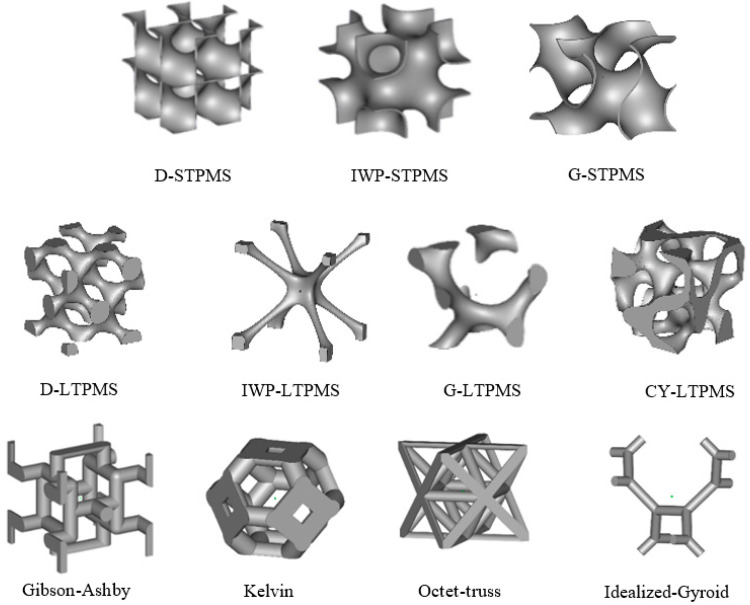
One-unit cell of different lattice architectural topologies: sheet-based TPMS (**top**), ligament-based TPMS (**middle**), and cellular struts (**bottom**).

Computer-aided design (CAD) files were created for different lattice architectures (see the unit cell of each lattice in Figure 1). The five strut-based lattices (Gibson–Ashby, Kelvin, Octet-Truss, and Idealized Gyroid) were designed using SolidWorks software (2014 Edition), and the files were exported in STL format for 3D printing. The TPMS lattices (three sheet-based lattices and four ligament-based lattices) were created using a software developed in-house called MSLattice [74], which generates STL files for 3D printing. TPMS topologies were generated using mathematical level set approximation equations [26,75] presented in Table 1:

(1) $$f\left(x,y,z\right)=c$$

where *c* is the level set parameter, which controls the solid volume fraction (or equivalently, the relative density) in the TPMS lattice; x=2πX/l, y=2πY/l, and z=2πZ/l, with l being the size of the unit cell; and X, Y, and Z are the Cartesian coordinate system. For c=0, the generated isosurface divides the space into two domains of equal volume.

Depending on the design approach, the TPMS-based lattices are either sheet-based or ligament-based [5,29]. The surface in the sheet-based TPMS (STPMS) is thickened to vary the relative density (RD) through $c\leq f\left(x,y,z\right)\leq c$. Alternatively, if one of the domains separated by the TPMS isosurface is solidified (i.e., f≥c or f≤c), the resulting topology is a ligament-based TPMS (LTPMS), and the RD or the volume fraction of the solid is specified by the value of c. In this study, LTPMS topologies are created through setting $f\left(x,y,z\right)\geq c$, where *c* is positive, such that $f\left(x,y,z\right)<c$ is the void space. Table 2 portrays *c* values and their corresponding RDs. The designed unit cells are tessellated equally in the 3D space with periodicity of 5×5×5 unit cells with 8 mm unit cell size. It is reported in [5,18] that 5×5×5 unit cells are sufficient to obtain effective (bulk) mechanical properties. The designed RD of the different lattices ranges between approximately 6% and 25%.

### 2.2. Experimental Methods

#### 2.2.1. Additive Manufacturing

**Figure 2:**
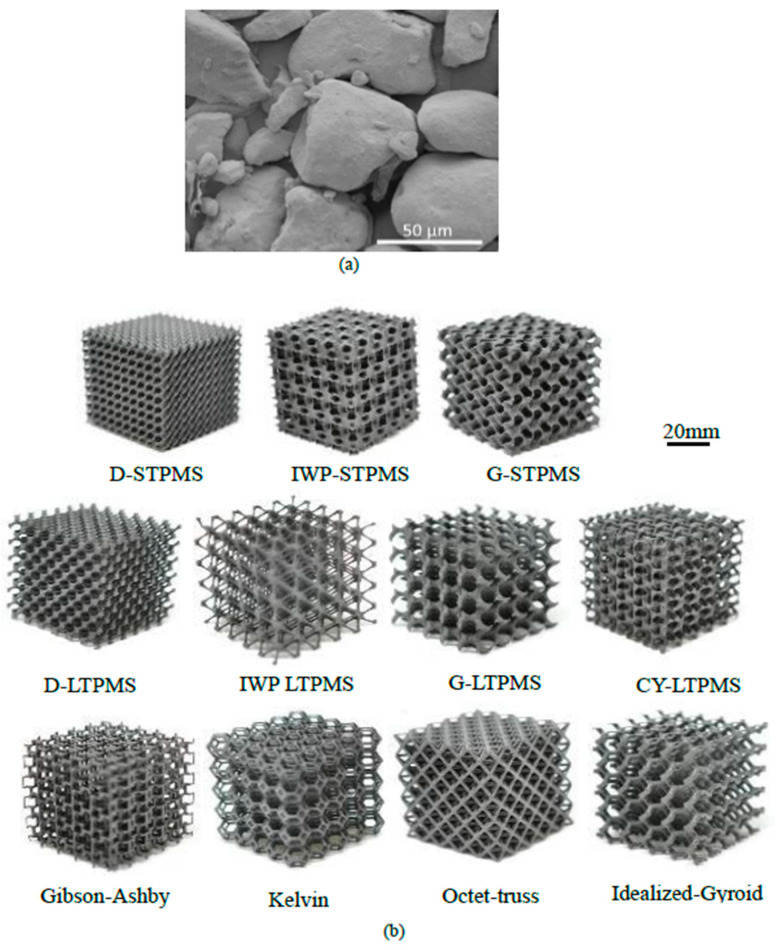
(**a**) SEM image of the shape and size of the base PA1102 black powder. (**b**) Samples 3D-printed by SLS technique of different cellular topologies; sheet-based TPMS (top), ligament-based TPMS (middle), and cellular struts (bottom) with 5 unit cell periodicity and 8 mm unit cell size.

The different polymeric cellular architectures were fabricated using the selective laser sintering (SLS) technique, also known as polymer laser powder-bed fusion, using the Formiga P110 3D printer by EOS GMbH (Munich, Germany). The base material used for 3D printing was PA1102 black, which is a mass-colored black polyamide 11 powder [76], with similar properties to Nylon. The typical powder size is roughly 50 μm, and Figure 2a portrays a SEM image of the powder particles. The printing parameters used in this study were 80 μm powder layer thickness, 21 W laser power, 200 μm laser hatch spacing, 2.5 m/s laser scan speed, and 170 °C powder bed temperature. Additionally, 40 mm cubic lattice structures were designed and fabricated with a 5×5×5 periodicity and 8 mm cell size at five different RDs. Two samples for each lattice were tested at each RD for repeatability and to ensure the reliability of the results. Figure 2b shows example optical images of 3D-printed samples.

#### 2.2.2. Architectural Characterization

After fabrication, architectural characterization was performed using the Quanta^TM^ 250 FEG scanning electron microscope (SEM) to evaluate the 3D printing resolution. Moreover, the interior structure of the fabricated lattices was scanned using a micro-computed tomography (CT) scanner (Nanotom m, General Electric (GE)), which has an ultra-high-performance nano-focus X-ray tube current with a wide sample and application range of 5–88 μA at 15 ISO, at an operating voltage up to 180 kV with the detail detectability of 0.2–0.3 μm. The CT scans were conducted at a high resolution of 22 μm per voxel, and two-dimensional (2D) 30 μm slice image data were collected. Visualization and reconstruction of the 3D designs of the fabricated lattices were executed with Avizo^®^ visualization software (2019 version).

#### 2.2.3. Mechanical Testing

**Figure 3:**
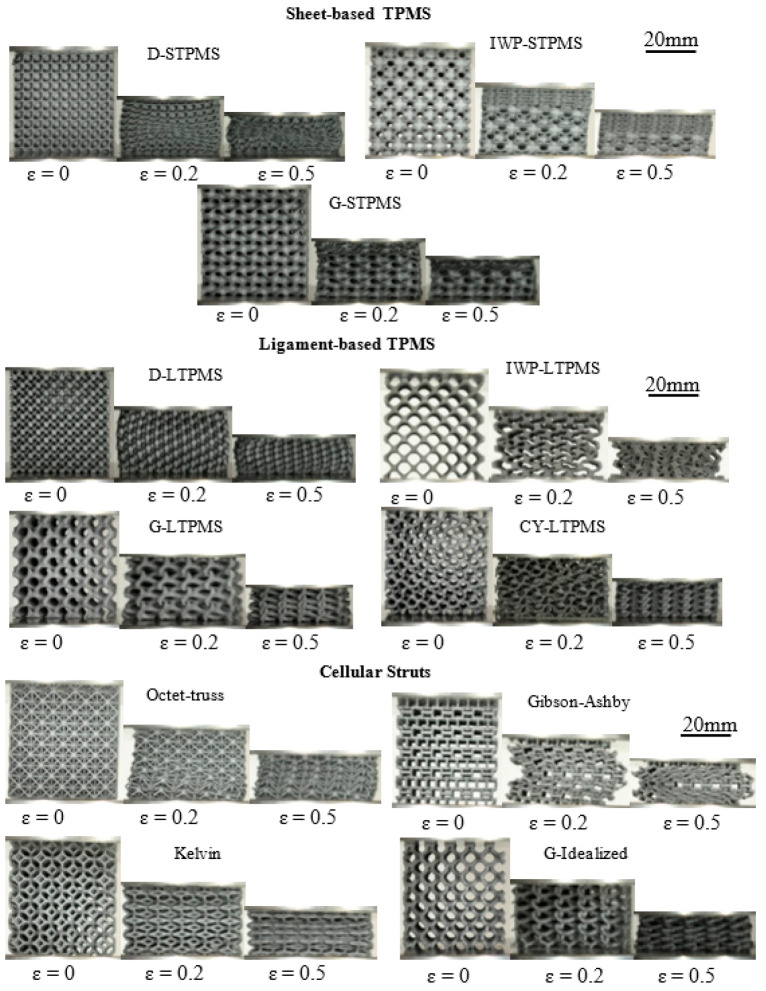
Deformation behavior of sheet-based TPMS, ligament-based TPMS, and strut-based lattices at different relative densities. The deformation behavior are presented at different strain levels;  ε=0.0, ε=0.2, and ε=0.5.

The compressive mechanical properties of the 3D-printed lattices were obtained by performing uniaxial compression tests using the Universal Material Testing Machine Instron 5980 Series with a 100 kN load cell and under displacement-controlled compression with a strain rate of 0.001/s. The displacement is attained from the perpendicular movement of the crosshead until 80% strain or full densification of the specimen, and the corresponding compression loading is measured. The subsequent deduced stress–strain responses were used to compute the uniaxial modulus, yield strength, compressive plateau strength, and toughness. Due to high replicability, only two samples of each lattice were 3D-printed and tested. The compression load was applied perpendicular to the printing direction. When 3D printing using the polymer SLS method, anisotropy in mechanical properties is minimal and can be ignored [18,76]. Figure 3 shows the deformation patterns under uniaxial compression of sheet-based TPMS, ligament-based TPMS, and strut-based lattices at different strain levels.

## 3. Results and Discussion

### 3.1. Assessing Morphology and Printability

**Figure 4:**
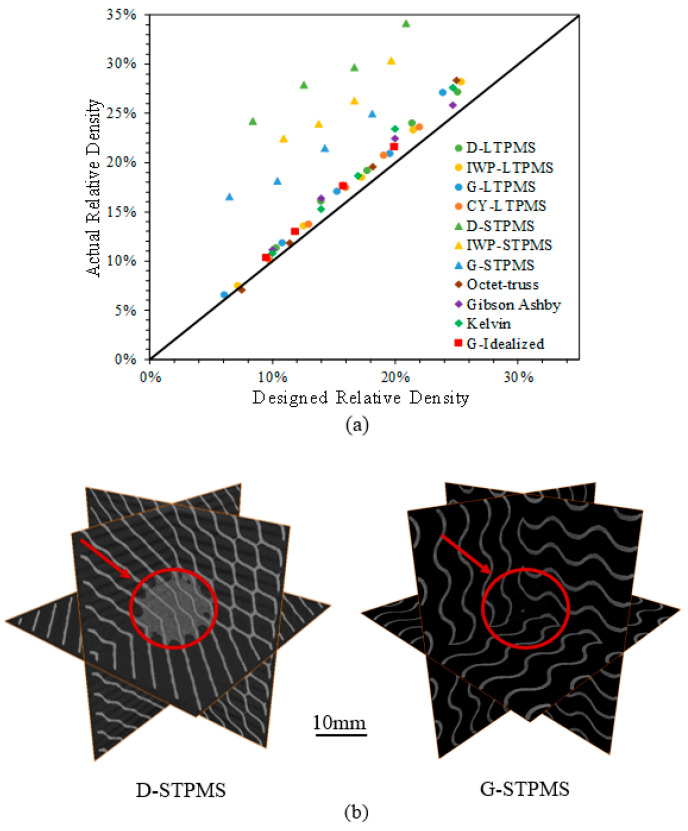
(**a**) Designed versus measured relative density for the sheet-based TPMS, ligament-based TPMS, and strut-based lattices. (**b**) CT scan images of 3D orthogonal slices showing the loose powder for the D-STPMS (8.4% designed RD and 24.2±1.7% measured RD) and G-STPMS (18.2% designed RD and 25.0±0.2% measured RD).

The designed RD is compared to the measured RD to assess the printability of the fabricated lattices. The measured RD is deduced by dividing the density of the lattice by the density of the base material, $0.99\;g/{\mathrm{cm}}^{3}$ [76]. The density of the lattice is calculated by air weighing the fabricated lattices and dividing the weight by the volume of the lattice. Figure 4a portrays the deviation of the designed RD and the 3D-printed measured RD for the lattice architectures. Table 3 and Table 4 show the difference between the designed RD and the measured averaged RD for strut-based and TPMS-based lattices, respectively. It can be observed from Figure 4a and Table 3 that the difference between the measured and designed RD for strut-based lattices is relatively small, between 0.2% and 3.4%. However, this is not the case for the sheet-based TPMS lattices (see Figure 4a and Table 4), which could be attributed to the intricate and complex structure where loose powder could become stuck in the interconnected pores, resulting in imprecise calculations of the actual RD, as shown through the CT images in Figure 4b for sheet-based Diamond TPMS lattice (D-STPMS).

It is also worth noting that the sheet-based lattices have higher surface area than strut-based lattices. In the context of the previous conclusion, more powder is most likely to fuse on the surface of the lattices. This could be attributed to the thermal gradient between the melted element and the loose powder nearby. We can say that the higher the surface area, the higher the deviation; this is also concluded by Al-Ketan et al. [5]. Additionally, it should be noted that at the same unit cell size, the main variation in the RD depends solely on the wall thickness of the sheet-based lattices or the ligament diameter of strut-based lattices. Thus, the minimum relative density is constrained to the minimal feature size, which is the minimum printable wall thickness or strut/ligament diameter. This is mainly controlled by the powder size and the laser spot size. The minimum feature size of the SLS printer is approximately 250 μm.

**Figure 5:**
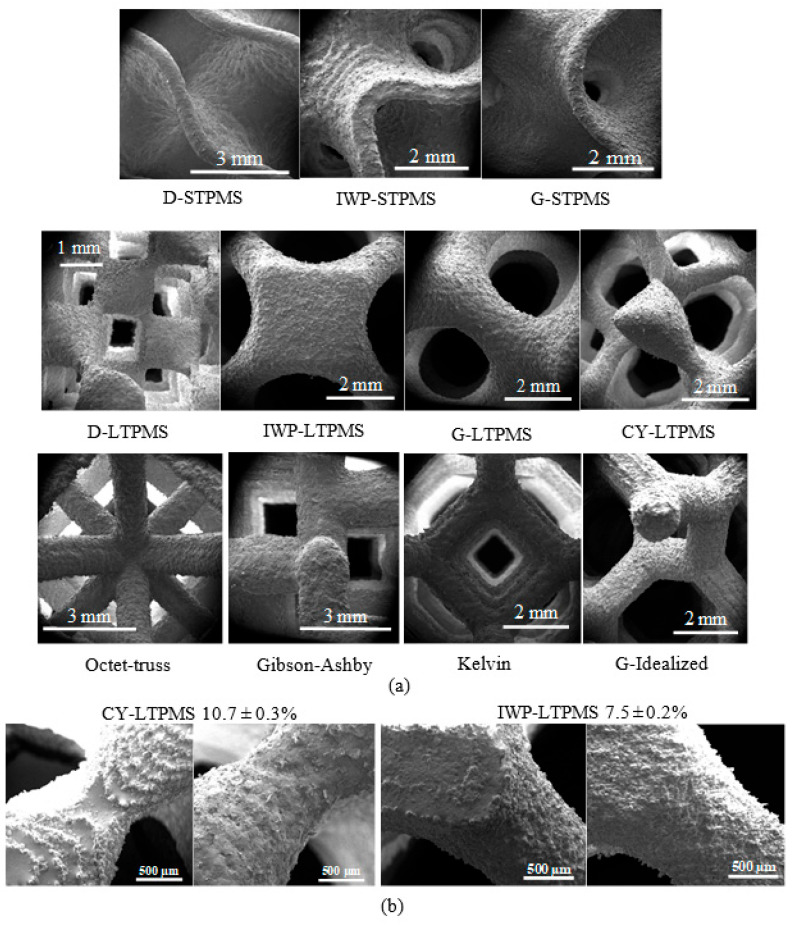
(**a**) Illustration of SEM images showing the printing quality of sheet-based TPMS lattices (top), ligament-based TPMS lattices (middle), and strut-based lattices (bottom). (**b**) SEM images showing the printing powder for the top (left) and bottom (right) views.

Examples of SEM images of all the topologies are shown in Figure 5a, with smooth intersections of the curves for the sheet-based and ligament-based TPMS lattices, whereas the intersections are sharp for the strut-based lattices. Generally, the SEM images denote high printing quality and manufacturability of the current lattices through the SLS 3D printing technique. There are several aspects that contribute to the presence of sticking powder on the surface; hence, the printing quality and morphology of the SLS fabricated topologies can be affected by the curved and inclined topologies; this increases the stair stepping effect [52]. The stepping effect can be observed in the top surface of the printed sample, as portrayed in Figure 5b, showing the difference between the printing surface of the top view and bottom view of the CY-LTPMS and IWP-LTPMS. The stepping effect can be reduced by decreasing the layer thickness; however, this increases the total fabrication time. The surface quality can also be affected by the thermal diffusion between the fused material and loose powder, causing powder to stick to the surface of the lattice, which is due to the significant difference in temperature [77]; the sticking powder on the bottom is shown in Figure 5b (right).

### 3.2. Compressive Mechanical Behavior

**Figure 6:**
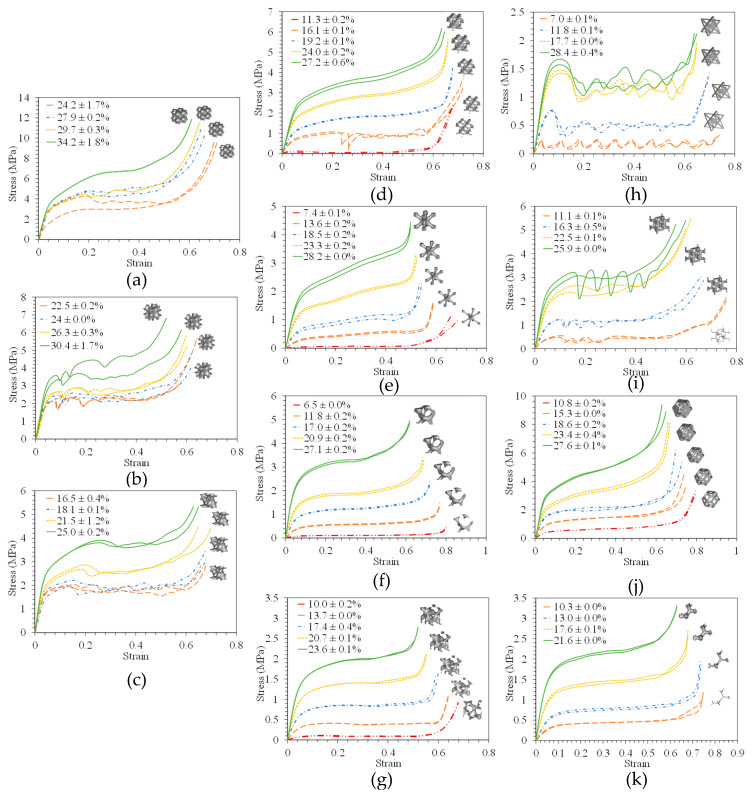
Stress–strain responses for the different lattice topologies of two replicates at various measured RDs: (**a**) D-STPMS, (**b**) IWP-STPMS, (**c**) G-STPMS, (**d**) D-LTPMS, (**e**) IWP-LTPMS, (**f**) G-LTPMS, (**g**) CY-LTPMS, (**h**) Octet-Truss, (**i**) Gibson–Ashby, (**j**) Kelvin, and (**k**) Idealized Gyroid. All samples are compressed at a strain rate of 0.001/s.

The effect of varying the relative density for each lattice on its uniaxial compressive behavior is comprehensively investigated experimentally. The compressive stress–strain responses at different RDs are portrayed in Figure 6. Overall, each lattice exhibited comparable responses with increasing mechanical properties while increasing RD. A typical cellular structure’s compressive stress–strain response is depicted [1]. The stress–strain curve is composed of an elastic region with a linear trend. The elastic range ends when the material reaches the yield strength where hardening starts, and it continues with a plateau stress until maximum strength. Finally, after reaching the maximum strength, the specimen starts to densify, and the densification region is the final region. Generally, the plateau region displays a smooth stress–strain curve without visible stress fluctuations, unlike the observations exhibited in the studies of Al-Ketan et al. [5] or Yan et al. [52], where the metallic D-LTPMS exhibited a fluctuating behavior in the plateau region. Al-Ketan et al. [5] reported that the D-LTPMS additively manufactured out of Maraging steel using selective laser melting (SLM) exhibited a significant drop in the stress–strain curve after reaching the maximum strength. This drop is attributed to the failure of the first layer of the topology. In fact, a similar behavior is depicted for the SLM AlSi10Mg D-LTPMS investigated by Yan et al. [52]. Conversely, the compressive properties of G-LTPMS were investigated using a range of materials, such as stainless steel [51], Maraging steel [5], and titanium alloy [22], and their stress–strain responses are in agreement with the behavior observed in the current study. Similarly, the compressive properties of the IWP-LTPMS were explored using Maraging steel [5] and titanium alloy [57], where small fluctuations in the stress–strain responses are portrayed particularly at high relative densities. Generally, fluctuations in the stress–strain curves can indicate the damage onset of some unit cells or the collapse of a layer of unit cells, whereas a smooth plateau stress might be an indication that the unit cells are experiencing plastic deformation without severe damage. On the other hand, the Octet-Truss lattice exhibited a fluctuating response, i.e., a drop at each layer after the failure of the previous layer; the same behavior was observed by Al-Ketan et al. [5]. Generally, the higher the relative density, the higher the slope of the plateau region (i.e., higher hardening). It is noteworthy that the two stress–strain curves for Gibson–Ashby lattice samples with RD of 25.9% differ significantly. This could be attributed to manufacturing defects, as we can conclude that one sample exhibits a layer-by-layer deformation, as evidenced by the fluctuation in the stress–strain response, whereas the other sample shows a smooth stress–strain curve, indicating collective plastic deformation of all layers, similar to LTPMS lattices. One can also see a collective deformation at 22.5% relative density, whereas all samples at relative densities of 16.3% and 11.1% show layer-by-layer deformation.

**Figure 7:**
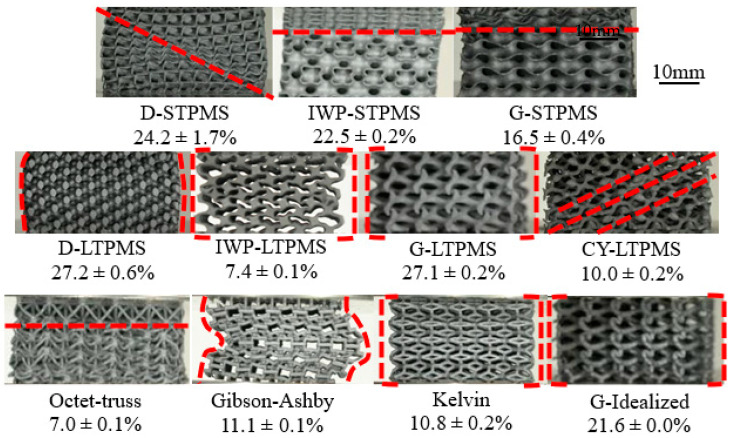
Deformation behavior of the various 3D-printed lattices at 50% strain level with their corresponding measured relative densities.

Taking a closer look at the deformation mechanism of the 3D-printed lattices, it is noted that the deformation pattern is influenced mainly by the lattice topology. The deformation patterns of the different lattices are presented in Figure 7. Although these deformation patterns are all portrayed at 50% strain, the measured relative density varies for each topology. Each topology at varying relative densities exhibited the same deformation pattern; thus, the deformation pattern depends significantly on the topological design and does not depend on the range of RDs considered in this work. The most observed deformation pattern is the collective plastic deformation, where the full lattice deforms altogether; this deformation is exhibited by the LTPMS, Kelvin, and Idealized Gyroid lattices. An additional observable deformation is the layer-by-layer failure. This is experienced by IWP-STPMS, G-STPMS, and Octet-Truss lattices. Gibson–Ashby lattice also exhibited an overall layer-by-layer deformation with global buckling. D-STPMS has a visible shear band deformation at a 45° angle, and after the shear, the layers began to fail in a diagonal direction, unlike metals, as Al-Ketan et al. stated that most of the topologies were deformed by shear bands [5]. As we emphasize in the following section, both topology and additive manufacturing defects are the main factors that determined the observed deformation behavior of the printed lattices.

### 3.3. Compressive Mechanical Properties

**Figure 8:**
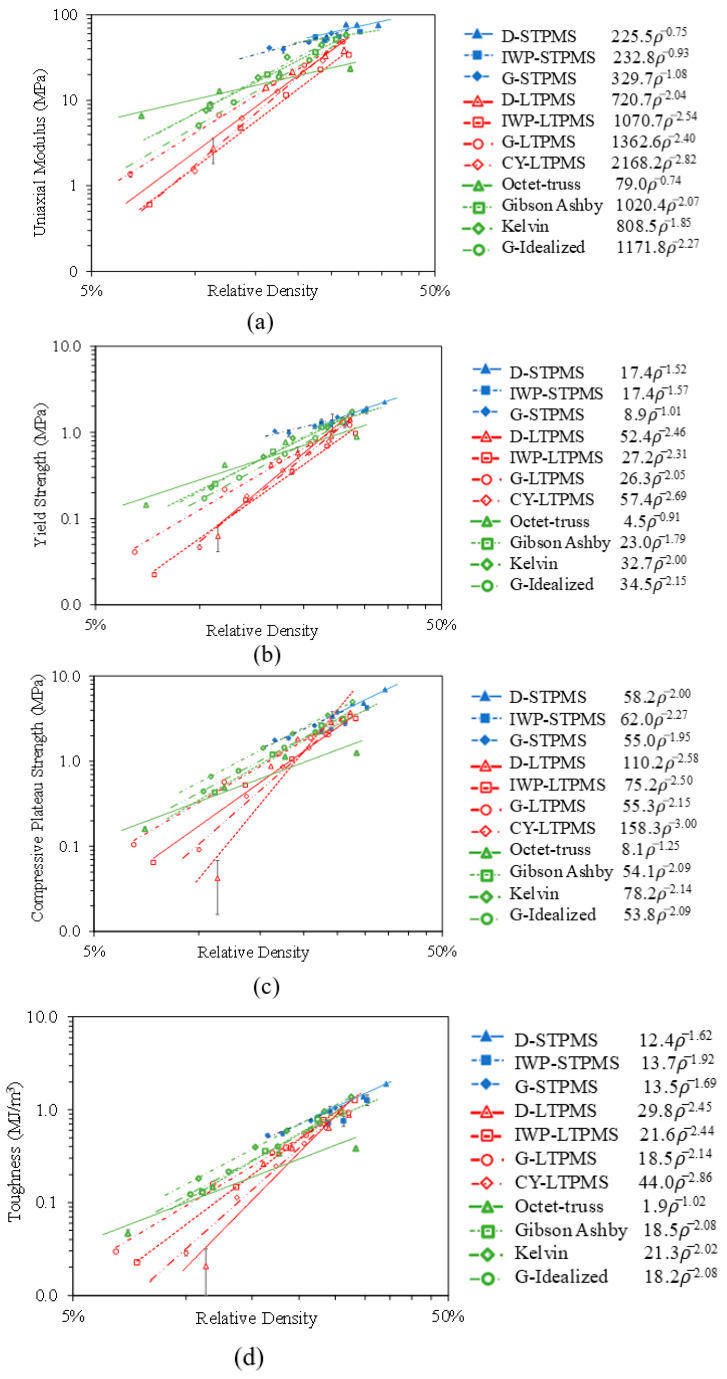
Deduced mechanical properties of different architectural topologies fitted with the Gibson–Ashby power scaling law: (**a**) uniaxial modulus, (**b**) yield strength, (**c**) plateau strength, and (**d**) toughness up to 40% strain.

The compressive mechanical properties of each lattice structure at each RD are compared with respect to the uniaxial modulus, yield strength, compressive plateau strength, and toughness, as illustrated in Figure 8. The uniaxial modulus is determined by the gradient of the linear elastic part of the stress–strain diagram before yielding, and the yield strength is computed by a parallel line to the gradient of the linear elastic part with a 1% strain offset [5,46]. The toughness (or commonly referred to as the energy absorption) is calculated as the area under the stress–strain curve up to 40%, and the compressive plateau strength is calculated as the mean of the stresses between 20% and 50% strain. The mechanical properties are plotted against RD in log-log scale graphs (see Figure 8). The graphs are fitted using the following Gibson–Ashby scaling power law [1]:

(2) $$M=C\;{\bar{\rho }}^{n}$$

where M is the normalized mechanical property; C is a geometrical parameter controlled by the lattice topology and solid along the loading direction, applied loading, manufacturing defects, and boundary conditions; $\bar{\rho }$ is the relative density; and n is the scaling exponent. The value of n can determine the different modes of deformation (stretching, bending, or mixed mode of deformation) of the cellular architectures [78]. Thus, n≈1 implies a stretching-dominated behavior, whereas n≥2 implies stiffness and n≥1.5 implies strength, indicate a bending-dominated deformation behavior; other values of n indicate a mixed mode of deformation. The fitting constants of the mechanical properties and scaling exponent n are listed in Table 5 based on the measured RD.

It can be noticed from Figure 8 that the sheet-based TPMS lattices are of interest due to their exceptionally high compressive properties with regard to the uniaxial modulus and yield strength when compared to the ligament- and strut-based lattices. This is in agreement with the results reported on metallic cellular lattices [4,5,79]. It is noteworthy that in terms of compressive strength and toughness, the Kelvin lattice exhibited similar mechanical properties to the sheet-based TPMS lattices. Another important observation is that the sheet-based TPMS lattices exhibit different deformation modes, a mixture of both, but mostly exhibited a stretching-dominated behavior.

Following the sheet-based TPMS lattices are the common strut-based lattices, where the cellular struts showed enhanced mechanical properties in comparison to the ligament-based TPMS lattices. In terms of stiffness and yield strength, the sheet-based TPMS and the Octet-Truss lattices exhibited the best mechanical behavior at low relative densities when compared to other lattice topologies. The *n* exponents of the uniaxial modulus for the D-STPMS and the Octet-Truss have the lowest values of 0.75 and 0.76, respectively, indicating much less dependency on RD. A comparable *n* exponent for the D-STPMS of 0.8 was attained by Bobbert et al. [56], whereas Al-Ketan et al. [5] achieved a lower *n* value of 0.5 for the D-STPMS and 1.23 for the Octet-Truss. The G-STPMS and IWP-STPMS have approximately the same *n* exponent of 1, where Abueidda et al. [64] concluded that the IWP-STPMS also experiences a stretching-dominated mode of deformation for the polymer PA2200 fabricated by selective laser sintering, as did Al-Ketan et al. [5] through selective laser melting (SLM). Conversely, the Kelvin topology had a mixed deformation mode since the value of the *n* exponent varies between 1 and 2, i.e., 1.85. The ligament-based TPMS, Gibson–Ashby, and Idealized Gyroid lattices exhibited a bending deformation mode since the value of the *n* exponent is more than 2. This is in contrast to the conclusions of the Maraging steel SLM-printed topologies [5], where the G-LTPMS and Gibson–Ashby lattices have *n* exponents of 1.68 and 1.62, respectively, implying a mixed deformation mode. Yan et al. [39] have stated that the G-LTPMS lattice has a mixed deformation mode and that the Gibson–Ashby lattice has a bending deformation mode for Ti-6Al-4V lattices fabricated by SLM. Nevertheless, previous studies have confirmed that the G-LTPMS is a bending-dominated lattice when 3D-printed out of polymer using the direct writing method [80], rubber-like material fabricated by SLS [32], calcium sulfate fabricated by laser powder bed fusion [35], and photopolymer resin material fabricated by stereolithography [32,35,46]. These anomalies can be attributed to the process-induced defects due to different printing techniques, different process parameters, different materials, and loose powder. In fact, it is possible to find loose powder on the walls of the topologies that do not contribute to mechanical properties and thus substantially influence the weight of the architecture and, consequently, the measured relative density.

In terms of the yield strength, the *n* value observed is higher than 1.5, implying that at yielding, the topologies adopted a bending-dominated deformation mode for all the architectures, apart from the G-STPMS and Octet-Truss lattices, which exhibited a stretching-dominated deformation mode behavior with an *n* value of approximately ≈1. As for the compressive strength, the Octet-Truss lattice is the only architecture that exhibited a stretching-dominated deformation mode, as the *n* exponent is equal to 1.25, while all the other architectures and even the sheet-based TPMS lattices exhibited a bending deformation mode, as all the *n* values are more than 1.5. This is in agreement with other studies on metallic TPMS lattices, where it was concluded that the compressive strength of the sheet-based TPMS lattices exhibits a bending-dominated mode of deformation for the Diamond, IWP, and Gyroid lattices [5]. Additionally, Abueidda et al. [64] showed the same mode of deformation for the IWP-STPMS for the PA 2200 polymer fabricated by selective laser sintering, while Kadkhoapour et al. [57] concluded that IWP-STPMS exhibits a stretching-dominated mode of deformation for the Ti6Al4V lattices fabricated by selective laser melting. Kelvin lattice experienced the highest compressive strength and almost has a similar behavior to the D-STPMS and G-STPMS.

**Figure 9:**
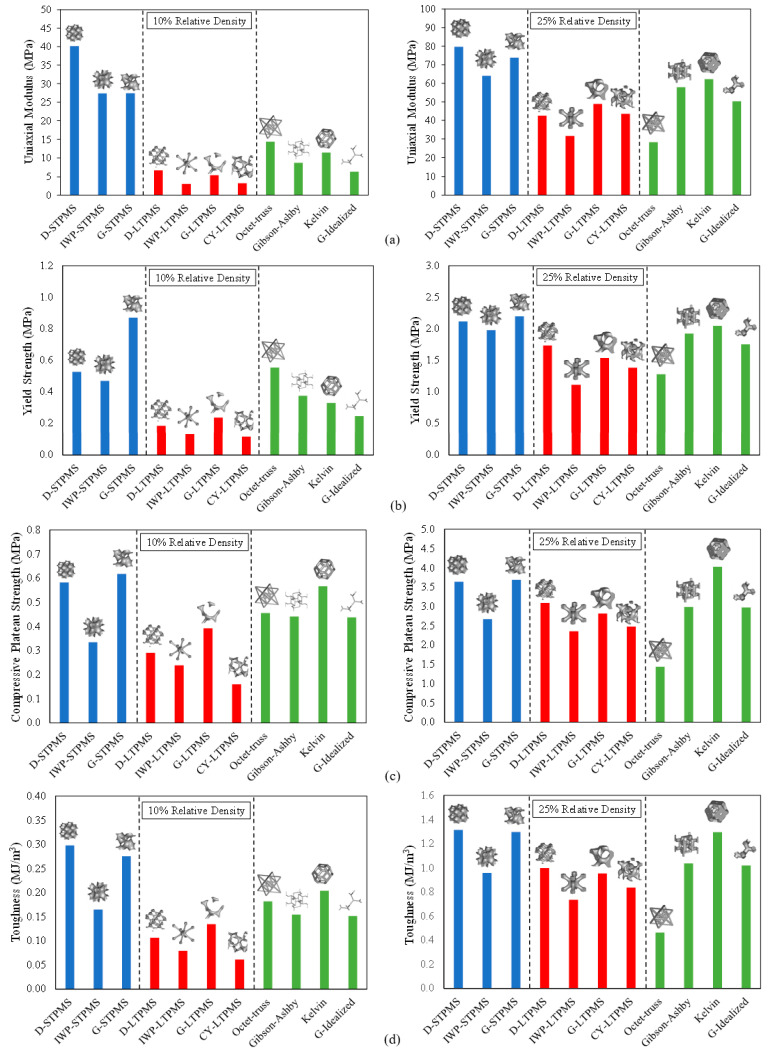
Compressive mechanical properties at 10% and 25% relative densities. (**a**) Uniaxial modulus, (**b**) yield strength, (**c**) plateau strength, and (**d**) toughness.

It is noteworthy that the toughness of bending-dominated lattices is usually larger than that of stretching-dominated lattices [78]. In fact, the Octet-Truss lattice exhibited low toughness when compared to the other lattices. On the other hand, it can be observed that the Kelvin lattice has a high toughness, unlike what has been reported for metallic sheet-based TPMS lattices [5] that exhibited significantly higher toughness than strut-based lattices, and the Kelvin lattice exhibited a lower toughness than the sheet-based TPMS lattices. Having the highest toughness implies suitability for energy absorbing applications with a better stiffness-to-weight ratio. Overall, it can be concluded that the effect of the lattice topology is stronger at low RD, and the mechanical behavior starts to converge at high relative densities. This is portrayed in Figure 9, which presents the compressive mechanical behavior of the topologies at fixed relative densities, specifically 10% and 25% to represent low and high relative densities. It can be seen that the effect of topology is more evident at low relative densities. In general, the sheet-based lattices have superior mechanical properties, and this is aligned with the results on metallic counterparts [5]. If the additive manufacturing technique allows the fabrication of lower relative densities (i.e., thinner sheets) for the sheet-based TPMS lattices, then the size effect might significantly enhance the mechanical properties. For example, Zheng et al. [81] fabricated micro-lattices based on cellular strut-based lattices, namely Kelvin and Octet-Truss, and were able to reach submicron size using the direct laser writing technique, coating the samples using electroless nickel plating, and then removing the polymeric substrate. This manufacturing method allows micro-scale unit cell fabrication with submicron features that utilize size effect and lead to enhanced mechanical attributes that are not achievable at macro-scales.

It is noteworthy that although the G-Idealized strut-based lattice is an idealization of the G-LSTPMS ligament-based lattice, overall, the G-Idealized lattice slightly outperforms the G-LSTPM lattice, especially at lower relative densities. Their elastic modulus and yield strength are almost the same, but the plateau strength and toughness of the G-Idealized lattice are slightly higher than that of G-LTPMS. In fact, one would expect G-LTPMS to perform better than G-Idealized due to its smooth surface with no geometric discontinuities. This counter-intuitive result can be mainly attributed to more manufacturing defects in G-LTPMS as compared to G-Idealized, as these defects affect the strength and toughness more than modulus and yield strength. Furthermore, while the results of the ligament-based TPMS lattices indicate that the G-LTPMS had superior mechanical properties among ligament-based lattices and is more promising at low relative densities, the D-LTPMS and CY-LTPMS had the lowest mechanical properties, which could be attributed to their smaller cross-sectional area at many junction points within the lattice, making them more prone to manufacturing defects.

### 3.4. Further Discussion

Generally, it can be noticed that at high relative densities, the compressive mechanical properties start to converge for different lattice topologies as the effect of the base material starts to dominate. Moreover, Kelvin lattice shows mechanical properties as high as the STPMS lattices at high relative densities. Although Kelvin lattice exhibits a bending-dominated deformation behavior, it can be observed that it has the highest compressive strength at high relative densities. This could be attributed to the hardening effect, which increases as the relative density increases, such that at high relative densities, a plateau in the stress–strain response is not observed and the strength continues to increase with inelastic deformation. On the other hand, the Octet-Truss lattice initially fails, and then the stress–strain response reaches a plateau. In fact, Kelvin lattice becomes much stronger at higher relative densities as the strut thickens and seems like it is converging similar to a closed unit cell, in that its resistance to load increases significantly and starts to behave like a sheet-based lattice. Based on the previous analyses of the mechanics of lattice materials, lattices with a stretching-dominated deformation behavior are more desirable in the design for additive manufacturing as they offer superior mechanical properties at the same relative density when compared to the bending-dominated topologies. Therefore, stretching-dominated lattices such as the Octet-Truss lattice have been a matter of extensive research [82,83,84,85,86,87,88,89]. However, the substantial nodal interconnections of stretching-dominated strut-based topologies introduce several manufacturing and functioning limitations. Therefore, we attempted to answer the question of whether adopting alternative lattice designs can result in increased geometrical efficiency by employing TPMS topologies that are essentially node-free, intersection-free, and have a smooth and continuous structure. Several studies concluded that TPMS structures have adequate fatigue resistance behavior as there are no stress concentrations at the point of junction due to their continuous curvature [40,56,79,90]. They are also suitable for bio-cell culture and penetration, as the structures have suitable flow permeability [20,66,91].

**Figure 10:**
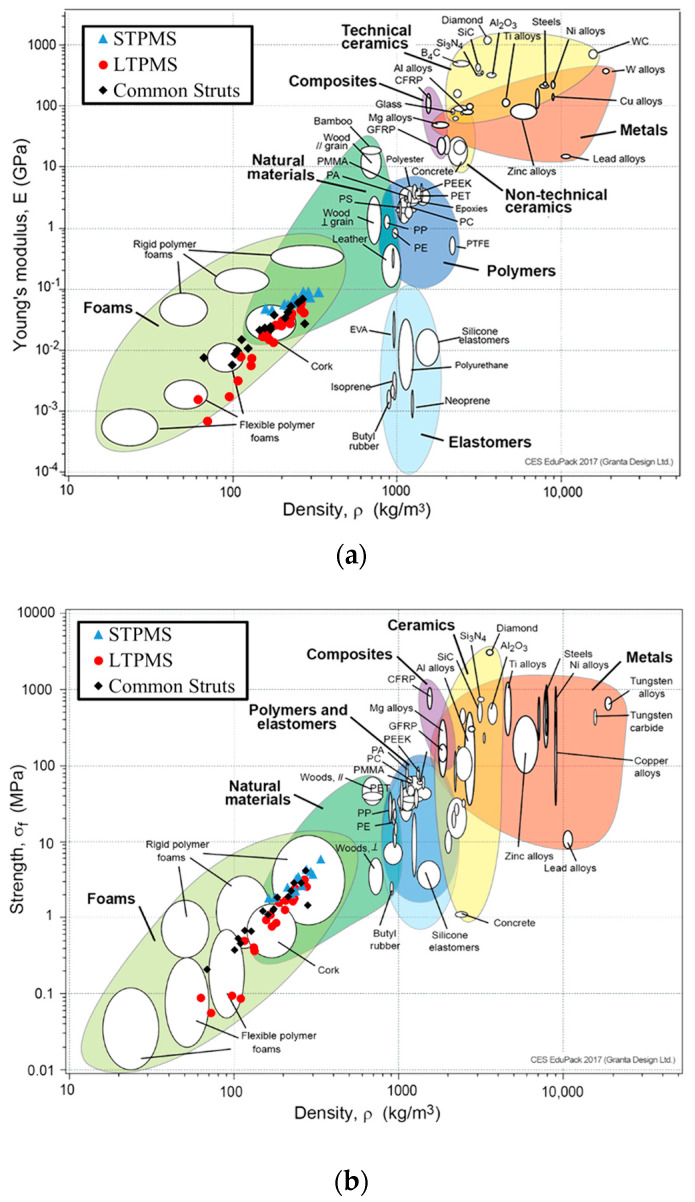
Ashby chart for different materials. (**a**) Young’s modulus versus density; (**b**) strength versus density (Generated by GRANTA EduPack software [92]).

The stiffness and strength of the lattice architectures tested here are compared with other materials in Figure 10a,b, respectively. The plots of the measured uniaxial modulus and compressive strength against the density for the different lattices (i.e., the ligament-based TPMS, sheet-based TPMS, and strut-based lattices) are also portrayed. The plots show that the mechanical properties of the investigated lattice architectures are located within the same class boundaries of the flexible polymeric foam cluster in the material property chart for engineering materials.

## 4. Conclusions

We comprehensively studied the topology–property relationship of three classes of architected cellular materials or metamaterials based on TPMS lattices of ligament (LTPMS) and sheet/shell (STPMS) types and other types of well-known strut-based lattices additively manufactured using polymer laser selective laser sintering (SLS). In total, 55 lattices (three STPMS, four LTPMS, and four strut-based lattices each of five relative densities) were 3D-printed and tested under quasi-static compressive loading. The printing quality of these lattices was evaluated using SEM and micro-CT. Furthermore, detailed comparisons were made between the extracted compressive mechanical properties (elastic modulus, yield strength, plateau strength, and toughness) of each lattice class of different topologies and relative densities. Overall, the results show that the stress responses of lattices 3D-printed using the SLS technique have smooth transition from elastic to plastic behavior, with a smooth hardening curve with increased deformation, except for the Octet-Truss lattice. Furthermore, STPMS lattices demonstrated a stretching-dominated elastic deformation behavior and a bending-dominated plastic behavior, and the latter deformation mode is mainly driven by manufacturing defects. Overall, however, STPMS lattices exhibited the best mechanical properties, with D-STPMS and G-STPMS having the best mechanical performance, followed by strut-based lattices and finally LTPMS lattices. Kelvin strut-based lattice exhibited high compressive strength and toughness that are comparable to STPMS lattices, especially at high relative densities. An interesting result is that the G-Idealized lattice, which is a strut-based idealization of the smooth G-LTPMS ligament-based lattice, exhibits slightly higher plateau strength and toughness than G-LTPMS at lower relative densities, which can be attributed to more manufacturing defects in the 3D printing of G-LTPMS.

Overall, the D-STPMS and Octet-Truss lattices exhibited the best compressive mechanical properties, as they are the least affected by the change in the relative density because of their small Gibson–Ashby *n* scaling exponents for various properties. However, it is noticed that at high relative density, the mechanical properties of all the topologies tend to converge, implying that the effect of topology is more significant at low relative densities. Furthermore, all the investigated ligament-based TPMS lattices demonstrated a bending-dominated deformation mode, indicating that they are best for energy absorption applications. This study emphasizes the attractive mechanical behavior of sheet/shell-based lattices as compared to strut/ligament-based lattices and their utilization in various applications.

## Figures and Tables

**Table 1:** Level set approximation equations of the TPMS topologies.

TPMS	Level Set Approximation
Schwarz Diamond 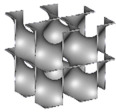	$\left(cosx\times cosy\times cosz\right)-\left(sinx\times siny\times sinz\right)=C$
Schoen IWP 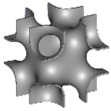	$2\left[\left(cosx\times cosy\right)+\left(cosy\times cosz\right)+\left(cosz\times cosx\right)\right]-\left(cos2x+cos2y+cos2z\right)=C$
Schoen Gyroid 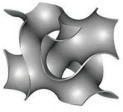	$\left(sinx\times cosy\right)+\left(siny\times cosz\right)+\left(sinz\times cosx\right)=C$
Fischer Koch-CY 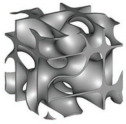	sinx×siny×sinz−sin2x×siny−sin2y×sinz−sin2z×sinx+cosx×cosy×cosz−sin2x×cosz−sin2y×cosx−sin2z×cosy=C

**Table 2:** Values of the level set constant *c* corresponding to each designed relative density (RD) for each TPMS lattice: sheet-based TPMS and ligament-based TPMS.

Sheet-Based TPMS Lattices							
D-STPMS			IWP-STPMS		G-STPMS		
RD (%)	*c*		RD (%)	*c*	RD (%)	*c*	
8.4%	−0.07 < *c* < 0.07		10.9%	−0.41 < *c* < 0.41	6.5%	−0.1 < *c* < 0.1	
12.6%	−0.11 < *c* < 0.11		13.8%	−0.52 < *c* < 0.52	10.4%	−0.16 < *c* < 0.16	
16.7%	−0.14 < *c* < 0.14		16.7%	−0.63 < *c* < 0.63	14.3%	−0.22 < *c* < 0.22	
20.9%	−0.18 < *c* < 0.18		19.7%	−0.75 < *c* < 0.75	18.2%	−0.28 < *c* < 0.28	
**Ligament-Based TPMS Lattices**							
**D-LTPMS**		**IWP-LTPMS**		**G-LTPMS**		**CY-LTPMS**	
**RD** **(%)**	***c***	**RD** **(%)**	***c***	**RD** **(%)**	***c***	**RD** **(%)**	***c***
10.3	0.67	7.2	2.79	6.1	1.30	9.8	1.76
14.0	0.61	12.6	2.57	10.8	1.16	13.0	1.63
17.8	0.55	17.3	2.35	15.3	1.04	16.0	1.50
21.4	0.49	21.5	2.11	19.6	0.92	19.1	1.37
25.1	0.43	25.4	1.89	23.9	0.80	22.0	1.25

**Table 3:** Designed versus measured RD for the strut-based lattices.

	Designed RD (%)	Measured RD (%)	Standard Deviation (%)
Octet-Truss	7.5%	7.0%	0.1%
	11.4%	11.8%	0.1%
	15.8%	17.7%	0.0%
	18.2%	19.6%	1.0%
	25.0%	28.4%	0.4%
Gibson–Ashby	10.0%	11.1%	0.1%
	14.0%	16.3%	0.5%
	20.0%	22.5%	0.1%
	24.7%	25.9%	0.0%
Kelvin	10.0%	10.8%	0.2%
	14.0%	15.3%	0.0%
	17.0%	18.6%	0.2%
	20.0%	23.4%	0.4%
	24.7%	27.6%	0.1%
Idealized Gyroid	9.5%	10.3%	0.0%
	11.9%	13.0%	0.0%
	15.8%	17.6%	0.1%
	20.0%	21.6%	0.0%

**Table 4:** Designed versus measured RD for the ligament-based and sheet-based TPMS lattices.

	Designed RD (%)	Measured RD (%)	Standard Deviation (%)
D-LTPMS	10.3%	11.3%	0.2%
	14.0%	16.1%	0.1%
	17.8%	19.2%	0.1%
	21.4%	24.0%	0.2%
	25.1%	27.2%	0.6%
IWP-LTPMS	7.2%	7.4%	0.1%
	12.6%	13.6%	0.2%
	17.3%	18.5%	0.2%
	21.5%	23.3%	0.2%
	25.4%	28.2%	0.0%
G-LTPMS	6.1%	6.5%	0.0%
	10.8%	11.8%	0.2%
	15.3%	17.0%	0.2%
	19.6%	20.9%	0.2%
	23.9%	27.1%	0.2%
CY-LTPMS	9.8%	10.0%	0.2%
	13.0%	13.7%	0.0%
	16.0%	17.4%	0.4%
	19.1%	20.7%	0.1%
	22.0%	23.6%	0.1%
D-STPMS	8.4%	24.2%	1.7%
	12.6%	27.9%	0.2%
	16.7%	29.7%	0.3%
	20.9%	34.2%	1.8%
IWP-STPMS	10.9%	22.5%	0.2%
	13.8%	24.0%	0.0%
	16.7%	26.3%	0.3%
	19.7%	30.4%	1.7%
G-STPMS	6.5%	16.5%	0.4%
	10.4%	18.1%	0.1%
	14.3%	21.5%	1.2%
	18.2%	25.0%	0.2%

**Table 5:** Fitting parameters of the power scaling law in Equation (1) for various compressive mechanical properties.

	Fitting Constants							
	Uniaxial Modulus (MPa)		Yield Strength (MPa)		Plateau Strength (MPa)		Toughness (MJ/m^3^)	
	*C*	*n*	*C*	*n*	*C*	*n*	*C*	*n*
D-LTPMS	720.7	2.04	52.4	2.46	110.2	2.58	29.8	2.45
IWP-LTPMS	1070.7	2.54	27.2	2.31	75.2	2.50	21.6	2.44
G-LTPMS	1362.6	2.40	26.3	2.05	55.3	2.15	18.5	2.14
CY-LTPMS	2168.2	2.82	57.4	2.69	158.3	3.00	44.0	2.86
D-STPMS	225.5	0.75	17.4	1.52	58.2	2.00	12.4	1.62
IWP-STPMS	232.8	0.93	17.4	1.57	62.0	2.27	13.7	1.92
G-STPMS	329.7	1.08	8.9	1.01	55.0	1.95	13.5	1.69
Octet-Truss	79.0	0.74	4.5	0.91	8.1	1.25	1.9	1.02
Gibson–Ashby	1020.4	2.07	23.0	1.79	54.1	2.09	18.5	2.08
Kelvin	808.5	1.85	32.7	2.00	78.2	2.14	21.3	2.02
G-Idealized	1171.8	2.27	34.5	2.15	53.8	2.09	18.2	2.08

## Data Availability

The data presented in this study are available on request from the corresponding author.
